# Direct Real-Time Neural Evidence for Task-Set Inertia

**DOI:** 10.1177/0956797614561799

**Published:** 2015-03

**Authors:** Lisa H. Evans, Jane E. Herron, Edward L. Wilding

**Affiliations:** Brain Research Imaging Centre, School of Psychology, Cardiff University

**Keywords:** task switching, episodic memory, task-set inertia, ERPs, recollection

## Abstract

One influential explanation for the costs incurred when switching between tasks is that they reflect interference arising from completing the previous task—known as task-set inertia. We report a novel approach for assessing task-set inertia in a memory experiment using event-related potentials (ERPs). After a study phase, participants completed a test block in which they switched between a memory task (retrieving information from the study phase) and a perceptual task. These tasks alternated every two trials. An ERP index of the retrieval of study information was evident in the memory task. It was also present on the first trial of the perceptual task but was markedly attenuated on the second. Moreover, this task-irrelevant ERP activity was positively correlated with a behavioral cost associated with switching between tasks. This real-time measure of neural activity thus provides direct evidence of task-set inertia, its duration, and the functional role it plays in switch costs.

Switching between different tasks is a common cognitive challenge. In the laboratory, task switching is usually examined by asking participants to switch frequently between trials associated with different tasks; on some trials, the task will change (*switch trials*), whereas on others it will not (*stay* or *repeat trials*). People are commonly slower and more error-prone on switch than on stay trials (Monsell, 2003). A central question in the task-switching literature relates to the identity and nature of the processes responsible for the costs involved in switching.

There are two main accounts of switch costs (for reviews, see Kiesel et al., 2010; Vandierendonck, Liefooghe, & Verbruggen, 2010). Central to both is the notion of task set, which is a collection of cognitive processes needed to perform the task. According to the *task-set-reconfiguration* explanation (Monsell, Yeung, & Azuma, 2000; Rogers & Monsell, 1995), the increase in reaction time (RT) on switch trials in comparison with stay trials reflects the time needed to engage control processes to reconfigure the cognitive system for a new task. An alternative account, *task-set inertia*, posits that the switch cost indexes the interference arising from the completion of a different task. Critically, this view emphasizes the difficulty in switching away from a task set, rather than in the time to prepare for an impending one. Allport and colleagues (Allport, Styles, & Hsieh, 1994; Wylie & Allport, 2000) argue that when individuals switch tasks, they still have activation persisting from the previous task set, and this can interfere with performance on the task at hand. The switch cost reflects the time needed to overcome this interference.

Empirical support for task-set inertia has been obtained from a variety of behavioral studies, in which persisting task set is inferred from the pattern of switch costs (see Kiesel et al., 2010). An alternative is to use techniques that arguably provide more direct insights into the processing of task-irrelevant information, such as eye movements and measures of neural activity. Longman, Lavric, and Monsell (2013) measured eye fixations in participants who were cued on every trial to identify a face or letters superimposed on it. On switch trials, participants fixated on the previously relevant feature to a greater extent than on stay trials. Moreover, there was a relationship between this attentional misorienting and switch costs. In functional MRI experiments, two different teams of researchers capitalized on the fact that distinct brain regions are engaged during the processing of certain stimuli (faces and words; Yeung, Nystrom, Aronson, & Cohen, 2006) or stimulus attributes (color and motion; Wylie, Javitt, & Foxe, 2006). Both teams reported increased activation of task-irrelevant information immediately following a task switch, and they also observed that the magnitude of this activation correlated with RT switch costs.

In the experiment reported here, we adopted the same approach as Longman et al. (2013) and Wylie et al. (2006) and cued our participants on a trial-by-trial basis so that we could determine more precisely the duration of task-irrelevant neural activity. There were also two important differences. First, one of our tasks did not rely on an assessment of perceptual characteristics of stimuli; instead, it required a memory judgment. This is an important departure, because in the three studies described previously, all switches were between perceptual elements (e.g., words or letters superimposed on faces); hence, conclusions about persisting activations encompassed only visible elements of a stimulus array (which are either task irrelevant or task relevant). Second, we employed event-related potentials (ERPs). In the context of a task requiring a memory judgment, ERPs offer opportunities to infer the engagement of specific processes on switch and stay trials because the functional significance of specific ERP modulations has been explored extensively.

Of particular relevance here is the fact that recollection—the recovery of specific details of episodes—has a distinct signature in the electrical record called the left-parietal ERP old/new effect. This effect consists of a relatively greater positivity for ERPs elicited by old rather than by new test stimuli attracting correct judgments. It is largest at left parietal scalp locations between 500 and 800 ms after stimulus presentation. The evidence linking this effect to recollection is substantial (for reviews, see Friedman & Johnson, 2000; Wilding & Sharpe, 2003), and the effect has been used in several studies as a marker of the extent to which recollection has occurred in the absence of converging behavioral evidence (Bergstrom, Velmans, de Fockert, & Richardson-Klavehn, 2007; Evans, Wilding, Hibbs, & Herron, 2010; Herron & Rugg, 2003; Mecklinger, Parra, & Waldhauser, 2009).

In the present experiment, participants completed an initial study phase followed by a test phase, during which ERPs were acquired. In the test phase, participants switched between an episodic memory task and a perceptual task; these alternated every two trials (Rogers & Monsell, 1995). If there was interference between tasks, the task set should carry over from the episodic memory task when participants complete the perceptual task. Evidence for this would be the presence of a left-parietal old/new effect, the neural index of recollection, in the perceptual task. Moreover, larger old/new effects on switch than on stay trials would provide information about the time course of this persistence. Finally, if task-set inertia plays a functional role in the switch cost, there should be a correlation between the degree of task-irrelevant activity and behavioral switch costs.

## Method

### Participants

Because of the novelty of this study, effect sizes could not be calculated straightforwardly to estimate sample size. Previous studies have demonstrated that robust left-parietal effects in memory tasks can be found with 16 participants (e.g., Wilding, Doyle, & Rugg, 1995; Wilding & Rugg, 1997). Anticipating somewhat smaller effects in the perceptual task here, as well as an assessment of changes in effects across switch and stay trials, we planned to include data from 32 participants.

Forty-eight right-handed native English speakers ages 18 to 30 years (35 females, 13 males) participated in the study for payment after giving informed consent. Sixteen were excluded: 11 failed to contribute sufficient artifact-free trials in the conditions of interest (i.e., ≥ 16), and 5 fell below the threshold for behavioral performance (proportion of correct location judgments given an “old” response < .6). Rules for the selection of participants were set a priori following standards adopted in our previous work (e.g., Evans et al., 2010). Of the 32 participants included, 24 were female, and 8 were male.

### Stimuli and design

Stimuli were 240 concrete nouns selected from the MRC Psycholinguistic Database (Coltheart, 1981) with Kucera-Francis frequencies of 1 to 9 per million. All words had between three and nine letters and were presented in Times New Roman font in white letters on a black background. The stimuli were presented on a monitor 1.2 m from the participant, and test stimuli subtended a maximum visual angle of 2.1° vertically and 2.5° horizontally.

The words were randomly assigned to 20 lists each containing 12 words. There were 10 study-test cycles. Within each cycle, one list was shown at study and again at test along with a second list. No lists were repeated across cycles. Half of the study words were presented on the left side of the monitor and half on the right, in a randomized order counterbalanced across participants. During the test phase, words were shown individually above, at, or below fixation, with an equal number at each location. Each of these words was preceded by one of two preparatory cues that indicated which task participants were to complete, and these were denoted by the capital letters “O” and “X.” The mapping of these letters to task was counterbalanced across participants. Each test-cue type was always presented for two consecutive trials.

The old/new status of words and the designation of words to the episodic or perceptual task were fully counterbalanced. At study, participants responded with their index and middle fingers, counterbalanced across left and right hand. Left-side location judgments were always associated with the leftmost of the two fingers. During the test phase, participants responded using the same fingers as at study, with the addition of the index finger of the other hand to indicate new words or words below fixation.

### Procedure

Each study-test block started with a message on screen indicating the number of the block participants were about to complete. At study, participants were asked to indicate whether a word appeared on the right or left side of the screen. A central fixation asterisk was presented for 1,000 ms, then a word was presented for 300 ms. The monitor then went blank until a response was made, after which the monitor remained blank for a further 500 ms before the start of the next study trial. Responses were made by pressing a key.

Each trial during the test phase started with a cue indicating which of two tasks participants should prepare to complete. In the episodic task, participants had to decide whether the word was new (i.e., not shown at study) or had appeared on the left or right side of the screen. In the perceptual task, participants had to indicate whether the test word had just appeared toward the top, middle, or bottom of the monitor. Each task required one of three responses: The episodic task required a “left,” “right,” or “new” response, and the perceptual task required a “top,” “middle,” or “bottom” response. The preparatory cue stayed on screen for 300 ms, followed by a central fixation asterisk for 2,000 ms, then the test word for 300 ms. The monitor then went blank until participants made a response, and it remained blank for a further 500 ms before the next preparatory cue was shown. Participants were asked to pay attention to the preparatory cue in order to identify the retrieval requirements and to respond accordingly. They were encouraged to balance speed and accuracy equally.

### Electroencephalogram (EEG) acquisition

EEG data were recorded during the test phase from 25 silver/silver-chloride electrodes embedded in an elastic cap and from two electrodes placed on the left and right mastoids. Recording locations were based on the international 10-20 system (Jasper, 1958, appendix) and included midline (Fz, Cz, Pz), fronto-polar (Fp1/Fp2), frontal (F7/F8, F5/F6, F3/F4), central (C7/C8, C5/C6, C3/C4), posterior (P7/P8, P5/P6, P3/P4), and occipital (O1/O2) sites. Vertical and horizontal eye movements were recorded from additional electrode pairs. EEG data were recorded at 167 Hz and referenced off-line to the average of the signal at the two mastoids. EEG and electrooculogram (EOG) data were recorded with a bandwidth from 0.03 to 40 Hz (−3 dB). Trials containing large EOG artifacts were rejected, as were trials containing analog-to-digital saturation or baseline drift exceeding ±80 µV. Other EOG blink artifacts were corrected using a linear regression estimate (Semlitsch, Anderer, Schuster, & Presslich, 1986). The researcher was blind to trial identity when processing EEG data. Procedures for the rejection of trials were set a priori and were based on standards we have adopted in previous work (e.g., Evans et al., 2010). Less than 5% of all trials were rejected because of EEG artifacts. The total epoch length was 1,536 ms with a 102-ms prestimulus baseline, relative to which all mean amplitude measures were taken. A seven-point binomially weighted smoothing filter (~22 Hz) was applied prior to analysis.

## Results

All analyses included the Greenhouse-Geisser correction for nonsphericity when necessary. Epsilon-corrected degrees of freedom are reported. A significance level of .05 was adopted for all analyses. The mean number of trials included in the averaged ERPs for each response category was as follows. For the episodic task, ERPs associated with correct location judgments to old words had a mean of 22 for switch trials (range = 16–29) and 21 for stay trials (range = 16–28); in addition, ERPs associated with new items that were correctly classified had a mean of 26 for switch trials (range = 19–30) and 26 for stay trials (range = 16–30). For the perceptual task, ERPs associated with correct location judgments for new words had a mean of 27 for switch trials (range = 16–30) and 29 for stay trials (range = 25–38), whereas for correct location judgments for old words, the mean was 28 for switch trials (range = 21–33) and 27 for stay trials (range = 19–30).^1^

### Behavioral data

In the study phase, the proportion of correct left/right judgments was at ceiling. For the episodic task, the likelihood of a correct “old” response to an old word, irrespective of the accuracy of location judgments, was greater than the likelihood of an “old” response to a new word for switch and for stay trials, *t*s(31) > 39.52, *p*s < .001, Cohen’s *d*_z_s > 6.99, Hedges’s *g*_av_s > 9.95 (Lakens, 2013). The probability of “old” responses to old words was .93 for both switch trials (95% confidence interval, or CI = [.91, .95]), and stay trials (95% CI = [.90, .96]), and the probability of correct responses to new words was .91 for both trial types (95% CI = [.88, .94]). The mean accuracy of location judgments was .79 (95% CI = [.75, .83]), and .78 (95% CI = [.74, .82]), for switch and stay trials, respectively, which was reliably above chance, *t*s(31) > 13.49, *p*s < .001, Cohen’s *d*_z_s > 2.39, Hedges’s *g*_av_s > 4.71. Accuracy in the perceptual task was close to ceiling: The mean proportion of correct responses for all trial types ranged from .95 to .98 (95% CI = [0.93, 0.99]).

**Fig. 1.**
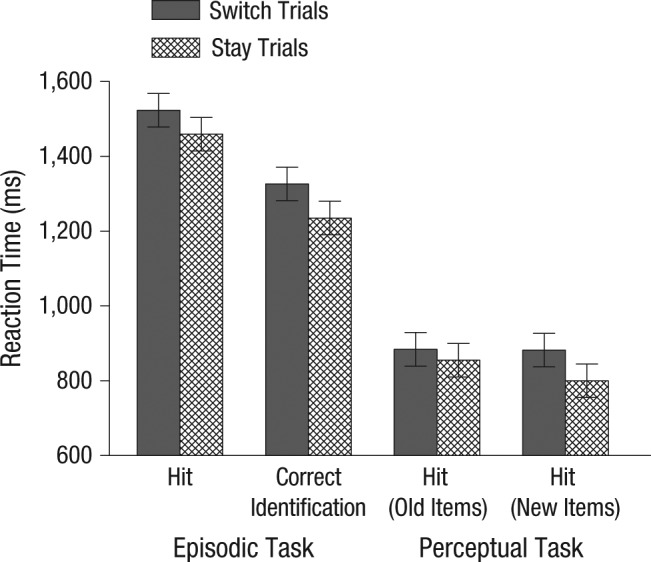
Mean reaction time as a function of task and trial type. For the episodic task, results are shown separately for correct location judgments (hits) and for correct identifications of new test items. For the perceptual task, results are shown for correct location judgments for previously studied words (old items) and for unstudied words (new items). Error bars indicate within-subjects confidence intervals (Loftus & Masson, 1994).

An analysis of variance (ANOVA) on RT data was conducted with the factors task (episodic, perceptual), trial type (switch, stay), and word status (old, new). RTs were slower on switch than on stay trials, *F*(1, 31) = 17.96, *p* < .001, ω_*p*_^2^ = 0.35. In addition, the Task × Word Status interaction was significant: RTs were faster in the perceptual task than in the episodic task, *F*(1, 31) = 148.74, *p* < .001, ω_*p*_^2^ = 0.82, and faster for new than for old words, *F*(1, 31) = 19.13, *p* < .001, ω_*p*_^2^ = 0.36, with the difference in RTs for word status being larger in the episodic than in the perceptual task, *F*(1, 31) = 18.09, *p* < .001, ω_*p*_^2^ = 0.35. These data are presented in Figure 1.

### ERP analyses

The neural index of recollection was measured between 500 and 800 ms after stimulus presentation. We ran a series of ANOVAs on the electrophysiological data, using the factors anterior-posterior dimension (anterior, central, and posterior), hemisphere (left, right), and site (inferior, midlateral, and superior) alongside factors of trial type (switch, stay) and word status (in the perceptual task: old, new; in the episodic task: correct location judgment, correct identification of new word).

First, analyses were conducted for the ERPs elicited in the perceptual task. At issue was how ERPs diverged on switch and stay trials when they were separated according to the task-irrelevant old/new stimulus dimension. The second set of analyses was conducted on ERPs elicited in the episodic task, in which the old/new dimension was relevant. In the following sections, only reliable effects involving word status are described. Finally, a correlation was computed between task-irrelevant activity on the perceptual task (i.e., the left-parietal ERP old/new effect) and the behavioral switch cost.

#### Perceptual task

**Fig. 2.**
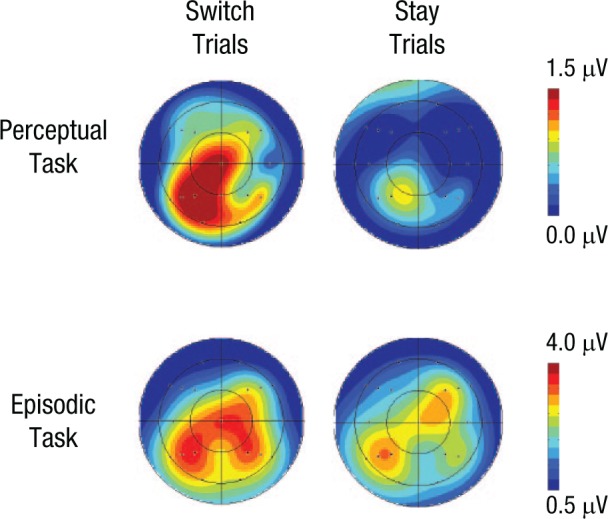
Scalp maps showing the distribution of old/new effects and their magnitude in the 500- to 800-ms time window after stimulus presentation. Results are shown separately for switch and stay trials in each of the two tasks. The old/new effect was calculated by subtracting the mean amplitude of event-related potentials (ERPs) elicited by new (i.e., unstudied) items from the mean amplitude of ERPs elicited by old (i.e., previously studied) items.

For the perceptual task, the scalp maps in Figure 2 show that the differences between activities in response to old and new words were somewhat left-lateralized, larger at posterior than at anterior scalp locations, and larger on switch than on stay trials. Statistical analyses revealed a Trial Type × Word Status × Anterior-Posterior Dimension × Hemisphere interaction, *F*(1.6, 48.3) = 3.79, *p* < .05, consistent with data shown in Figure 2.

**Fig. 3.**
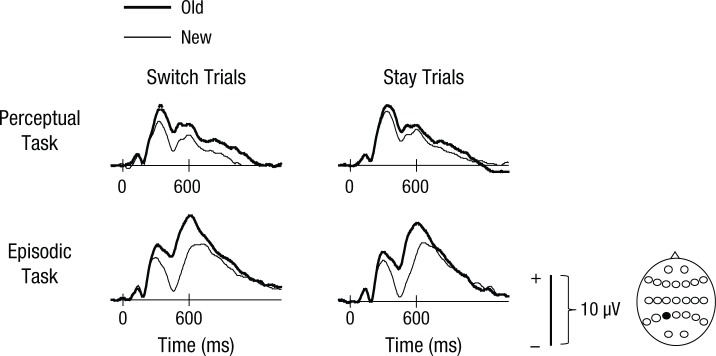
Grand-average event-related potentials (ERPs) from a left posterior superior electrode site (P3; indicated by the head diagram at the bottom right) for the perceptual and episodic tasks. For the perceptual task, waveforms are shown separately for switch and stay trials on which the locations of old and new words were reported correctly. For the episodic task, waveforms are shown separately for switch and stay trials on which the locations of old words were retrieved correctly and new words were identified correctly.

We followed up on this interaction by assessing switch and stay trials separately. There were no significant differences between ERPs elicited by old and new words on stay trials.^2^ On switch trials, there was a four-way interaction between word status, anterior-posterior dimension, hemisphere, and site, *F*(3.4, 104.6) = 3.32, *p* < .05, as well as two lower-order effects: a Word Status × Site interaction, *F*(1.1, 35.0) = 5.18, *p* < .05, and a main effect of word status, *F*(1, 31) = 6.21, *p* < .05. Examining ERPs elicited by switch trials at left posterior sites, where effects were largest, we found a main effect of word status, *F*(1, 31) = 12.13, *p* < .01, and an interaction with site, *F*(1.6, 49.0) = 9.77, *p* < .01. These results indicate that waveforms were more positive-going for old words than for new words at left posterior sites, particularly at sites closest to the midline (see Fig. 3).

For switch trials at one left posterior superior site (P3), there was a mean amplitude difference between responses to old words and responses to new words of 1.65 µV, *t*(31) = 3.72, *p* < .001, Cohen’s *d*_z_ = 0.66, Hedges’s *g*_av_ = 0.37. For comparative purposes, these same analyses were also conducted for stay trials, in which the marginal effect seen in Figures 2 and 3 was also largest at the P3 site. Here, the mean difference between ERPs in responses to old and new words was 0.85 µV, *t*(31) = 1.65, *p* > .1, Cohen’s *d*_z_ = 0.29, Hedges’s *g*_av_ = 0.17.

#### Episodic task

Figure 2 shows that the distributions of old/new effects in the episodic task were somewhat comparable on both trial types, but it appears that the magnitude was larger on switch than on stay trials. However, statistical analyses did not reveal reliable interactions involving the switch-versus-stay manipulation. There were interactions involving word status, anterior-posterior dimension, and site, *F*(2.4, 73.0) = 4.75, *p* < .01, and word status, anterior-posterior dimension, and hemisphere, *F*(1.2, 38.0) = 10.68, *p* < .01, as well as lower-level interactions involving these factors. Examining left posterior scalp locations revealed a main effect of word status, *F*(1, 31) = 58.35, *p* < .001. These results reflect the greater relative positivity for correct location judgments compared with correctly classified new test items at left posterior scalp locations. These can be seen clearly in Figures 2 and 3.

#### Task-set inertia and the switch cost

For each participant, the magnitude of the left-parietal old/new ERP effect (i.e., old items – new items) in the perceptual task was calculated for switch and stay trials separately. These data were taken from P3, where the effect was largest (see results for the episodic task). To calculate the degree to which this effect was reduced on switch trials relative to stay trials, we divided the magnitude of the effect on switch trials by the magnitude of the effect on stay trials. This ratio was then correlated with the behavioral RT switch cost observed in the perceptual task (indexed by dividing the RT on switch trials by the RT on stay trials).^3^ There was a significant positive correlation between the ERP and behavioral ratios, *r*(30) = .45, *p* < .02: Larger switch costs arose when there was a bigger reduction in the size of the old/new effect from switch to stay trials.

## Discussion

We used a novel approach to examine the presence of task-set inertia in a memory-task-switching experiment. We capitalized on the finding that there is an ERP index of the successful recovery of contextual information—the left-parietal old/new effect. This neural index was present in responses during our perceptual task, even though the study history of words was irrelevant for completion of this task. Notably, the left-parietal old/new effect was larger on trials in which participants had just switched from the episodic memory task than on trials in which participants had previously completed the perceptual task. Moreover, the pattern of neural activity differentiating responses to old and new words on switch trials in the perceptual task was similar to that found on switch as well as stay trials in the episodic task. This outcome is consistent with the view that processes engaged on the preceding task carried over and remained active on at least the first trial of the subsequent task. In a further analysis, we found that the magnitude of the left-parietal old/new effect in the perceptual task was related to the behavioral switch cost. This indicates that a reduction in the degree of task-irrelevant activity between switch and stay trials is associated with quicker RTs on stay than on switch trials. This finding is important because it demonstrates that the recovery of task-irrelevant information, which is presumably a consequence of task-set inertia, has a functional role in the switch cost. It is not the case that recollective activity runs in parallel to perceptual processing without influencing performance on the perceptual task.

It is clear that processes relevant to the episodic task were active during the perceptual task. Because of the timing and scalp topography of the effect seen in the perceptual task, together with the similarities to the effect observed in the episodic task, it seems reasonable to suggest that what is being carried over is the recollection of contextual information. Moreover, it seems likely that this information is the location of the word on the screen from the preceding study phase. As noted in the introduction, ERP studies of memory retrieval are now sufficiently advanced that the left-parietal old/new effect has been employed as a marker of recollection in the absence of behavioral data. For example, in a study by Bergstrom et al. (2007), participants initially learned a list of weakly associated word pairs. In the test phase, they saw the first word from the pair and were asked either to recall the associated word or to prevent the associated word from entering consciousness. On the basis of the magnitude of the left-parietal old/new effect, the authors were able to draw conclusions about the ability of participants to successfully avoid recollection.

Our study replicates Wylie et al.’s (2006) and Yeung et al.’s (2006) findings that task-irrelevant indices of neural activity are evident on switch trials and that there is a relationship between this persistence and behavioral switch costs. However, our study extends these findings in three important ways. First, the carryover effects reported here were not restricted to different elements or attributes of a visual stimulus. Second, they develop the important link between cognitive control and episodic memory (see Richter & Yeung, 2014). Third, they permit inferences about carryover at the level of a specific memory process. The findings also complement a recent behavioral study by Richter and Yeung (2012) examining the relationship between task switching and memory. In that study, participants saw words superimposed on pictures and were cued on each trial to attend to one or the other. Their memory was then tested for the words and the pictures. Task switching improved memory for task-irrelevant information: Participants remembered more about the unattended elements when they had been presented on switch rather than stay trials. This study and the current one illustrate that there is interdependence between task switching and episodic memory, which is rarely studied, but could potentially help to elucidate the mechanisms involved in each.

A notable element of the findings reported here was the absence of marked differences between the magnitudes of old/new ERP effects in the episodic task on switch and on stay trials. It might be anticipated that task-set inertia would affect the processing of both tasks, whereas this was only true for the perceptual task. One factor that was likely important in this respect is differences in task difficulty. Previous work has demonstrated that carryover effects are affected by the relative difficulty of the two tasks (Allport et al., 1994; Monsell et al., 2000; Yeung & Monsell, 2003). Switch costs are usually higher for the easier, more dominant task, as the increased control required to perform the hard task has more carryover than the control required for the easy task. This seems to fit with the pattern of ERP findings reported here. The lack of a difference in the magnitude of the left-parietal old/new effect between switch and stay trials in the episodic task may simply be due to the fact that the perceptual task had limited carryover, as it was substantially easier than the memory task.

In conclusion, strong evidence for task-set inertia was found only on the first trial after a cue to switch tasks. There was also compelling evidence that this carryover plays a functional role in the switch cost, as participants who recovered more task-irrelevant material exhibited larger behavioral switch costs. This insight was made possible by using an approach that is novel in the task-switching domain. We believe this paradigm and technique can be developed further to address new and fruitful questions in the areas of cognitive control as well as episodic memory.

## Notes

1. All participants received the same number of old and new items in the perceptual task; however, for 3 participants, these items were distributed slightly unequally between switch and stay trials.

2. The interaction between word status, anterior-posterior dimension, and site was just outside of the significance level adopted, *F*(2.9, 90.3) = 2.74, *p* = .05.

3. The correlation was also significant if a subtraction measure was used to calculate the behavioral switch cost, *r*(30) = .37, *p* < .05.
